# Familial Adenomatous Polyposis—Rendering a Diagnosis Based on Recognition of an Unusual Primary Thyroid Neoplasm

**DOI:** 10.1155/2011/767610

**Published:** 2011-03-02

**Authors:** David F. Schaeffer, Eric M. Yoshida, David A. Owen, Kenneth W. Berean

**Affiliations:** ^1^Department of Pathology and Laboratory Medicine, The University of British Columbia, Vancouver, BC, Canada V6T 1Z4; ^2^Department of Pathology, Vancouver General Hospital, 855 West 12th Ave, Vancouver, BC, Canada V5Z 1M9; ^3^Department of Medicine, The University of British Columbia, Vancouver, BC, Canada V6T 1Z4

## Abstract

It has been well established in the literature that the cribriform-morular variant of papillary thyroid carcinoma (CMVPTC) has been observed with higher frequency in familial adenomatous polyposis (FAP) patients. In the usual setting, patients with FAP are identified based on their germline mutations and the diagnosis of thyroid neoplasm is made after the FAP diagnosis. We herein report a case in which the recognition of a CMVPTC led to the initial diagnosis of FAP. The histological and clinical features of CMVPTC are reviewed with emphasis on its relationship to FAP.

## 1. Introduction

Thyroid neoplasms occur in patients with familial adenomatous polyposis (FAP) with a frequency reported to be increased to as much as 100–160 times the rate seen in the general population [1, 2]. Harach et al. [3], in a report of 28 tumors in 4 patients with FAP, were the first to recognize and describe in detail the unusual morphology of these tumors. They suggested that FAP-associated thyroid tumors have similarities to typical papillary thyroid carcinoma (PTC), but are sufficiently unique that they should be considered a tumor distinct from both follicular and papillary carcinoma. There are two reported cases in the literature of recognition of this unusual thyroid neoplasm leading to the diagnosis of familial polyposis [4]; sporadic counterparts, non FAP associated, have been described as well [5]. We herein describe a case of diagnosis of FAP in an individual without a family history of colonic disease based on recognition of a thyroid neoplasm of unusual morphology.

## 2. Case Report

A 23-year-old Chinese woman underwent total thyroidectomy for a painless enlargement of the thyroid gland. The enlargement had first been noted 10 years prior to admission, and there had been a gradual increase in size during the intervening period. The patient had no family history of colorectal cancer and both parents were in good health. She did not have any gastrointestinal symptoms and was otherwise well. A total thyroidectomy was performed for a clinical diagnosis of nodular goitre.

On the basis of the thyroid pathology, a flexible sigmoidoscopy was performed. This revealed numerous small (<5 mm) polyps scattered in clusters from rectum to descending colon. Biopsies of these polyps showed that they were all tubular adenomas. At colonoscopy there were more than 100 small polyps throughout the colon but predominantly left-sided. There were no polyps found on esophagogastroduodenoscopy. DNA extracted from blood lymphocytes showed a truncating mutation in segment 1 of the *APC* gene by protein truncation analysis, confirming the diagnosis of FAP. A total colectomy was performed.

One year following colectomy, two firm, immobile intraabdominal masses were noted on physical examination. An ultrasound directed biopsy of the masses revealed fibromatosis.

## 3. Materials and Methods

The thyroidectomy specimen was fixed in formalin, and representative paraffin-embedded sections were routinely stained with hematoxylin and eosin. Immunohistochemical staining was performed with the labelled streptovidin-biotin-peroxidase detection system using a panel of antibodies (see Table 1).

### 3.1. Pathology

The excised thyroid weighed 72 g and contained one nodule (5 × 4 × 4 cm) in the right lobe and three nodules in the left lobe ranging in size from 0.6 × 0.6 × 0.5 to 3 × 2.5 × 2.0 cm.

**Figure 1:**
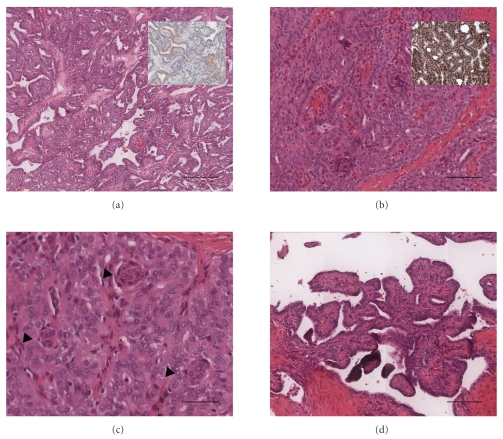
Histological features of CMV variant of PTC. Representative pictures of the tumour show the cribriform structures composed of follicles deficient in colloid and lined by flattened to low cuboidal epithelial cells with scant intervening fibrous tissue (a). The tumor cells were weakly positive by immunohistochemistry for thyroglobulin ((a)-inset), but showed nuclear localization with immunostaining for beta-catenin ((b)-inset). Other portions of the tumor nodules had a trabecular arrangement (b, c) of tall columnar cells with small whorls of cells (arrowheads). The cells within the whorls had nuclei with a pale eosinophilic to clear appearance (c). A minor papillary component was present within the periphery of the tumor nodule (d). The tumor cell nuclei were oval and overlapping with irregular outlines. Nuclear grooves were readily identified and occasional typical intranuclear cytoplasmic pseudoinclusions were present. Magnification bar: (a) ×40, (b & d) ×100, and (c) ×200.

Light microscopic examination revealed that the three nodules in the left lobe were surrounded by fibrous capsules of varying thickness. The architectural patterns within the nodules were variable, but papillae were present at least focally in each tumor. The papillae were generally short and blunt with edematous cores. A prominent finding within all of the nodules were cribriform structures composed of follicles deficient in colloid and lined by flattened to low cuboidal epithelial cells with scant intervening fibrous tissue (Figure 1). Other portions of the tumor nodules had a trabecular arrangement of tall columnar cells. Frequently within these trabecular areas and to a lesser extent elsewhere, there were small whorls of cells (Figure 1(c)). The tumor cell nuclei were oval and overlapping with irregular outlines. Nuclear grooves were readily identified and occasional typical intranuclear cytoplasmic pseudoinclusions were present. Many of the cells within the whorls had nuclei with a pale eosinophilic to clear appearance (Figure 1(c)). There was no necrosis or mitotic activity present.

Immunohistochemical studies showed that the tumor cells were weakly positive for thyroglobulin (Figure 1(a) inset) and EMA and strongly positive for keratin, CAM 5.2, and CK7. Beta-catenin immunostaining demonstrated nuclear localization within the tumor cells (Figure 1(b) inset). There was no cytoplasmic reactivity for CK20, chromogranin, or S100. In all of the immunostained sections, including the negative controls, there was strong positivity within the tumor cell nuclei with the peculiar nuclear clearing described above. 

Microscopic examination showed that the mass in the right lobe was a follicular adenoma. 

## 4. Discussion

The extraintestinal associations of familial adenomatous polyposis (FAP) are wellknown and include osteomas, epidermoid cysts, fibromatosis, adrenal, biliary, liver, and brain neoplasms (some kindreds with Turcot's syndrome) [4] as well as thyroid carcinomas [2, 6]. The risk of development of thyroid carcinoma in FAP is not known with certainty. A report from a large FAP registry indicated that 1.2% of affected individuals develop thyroid carcinomas [7]. Two large studies of FAP patients found a relative risk of developing thyroid carcinoma of 7.6 [8] and 23 [4] compared to the general population. However, amongst females with polyposis, the risk has been estimated at 100–160 times greater than expected in the general population [1, 2]. Some authors have recommended that FAP patients be screened for the development of thyroid carcinoma [1, 2], but this is controversial [7].

The FAP-associated thyroid tumors reported in the literature have been variably diagnosed as papillary, follicular, or mixed papillary and follicular carcinomas [3]. Harach et al. [3] reported four cases of thyroid carcinoma in FAP patients with a review of the literature and noted the following unusual features:

1. female : male ratio of 8 : 1 (2 : 1 to 4 : 1 in sporadic PTC); other studies have found an even more striking female predominance (f : m—17 : 1) for this particular subtye [9, 10],
2. majority under age 30 ( most in 30–50 age group in sporadic PTC),
3. multifocality (differing from sporadic PTC multifocality-tumors encapsulated, circumscribed, differing architectural patterns), and
4. unusual histologic patterns: cribriform, solid, spindling, and whorls.

The authors concluded that FAP-associated thyroid carcinomas were likely related to PTC, but were sufficiently different to warrant status as a distinct entity.

Based on the distinct and unusual morphology of the thyroid tumors occurring in FAP patients, Harach et al. [3] suggested that the recognition of such a tumor should prompt investigation for FAP. The investigation in the case reported herein led to a diagnosis of FAP, supporting their recommendation. 

However, Cameselle-Teijeiro and Chan [11] described 4 cases with similar or identical morphology apparently occurring in patients without FAP. Others [5, 12] have also described similar tumors, again in patients without evidence of FAP. These tumors have been termed the “cribriform-morular” variant of PTC (CMV PTC) by Cameselle-Teijeiro and Chan in recognition of their two most prominent and unusual patterns. The cribriform areas are composed of anastomosing bars and arches of cells without intervening stroma with the follicular spaces devoid of colloid. The morulas are composed of spindled cells with “peculiar nuclear clearing.” These clear nuclei differ from both the optically clear nuclei and intranuclear pseudoinclusions more typically seen in PTC and consist of accumulations of biotin [11]. 

Harach et al. [3] speculated that the morphology of this apparently distinctive tumor might be related to the involvement of the *APC* gene in the pathogenesis. Several groups have analyzed *APC* gene mutations. Cetta et al. [13] analyzed four tumors from patients with known germline *APC* mutations. They found no examples of biallelic inactivation. Soravia et al. [14] studied 9 samples from 4 tumors and found somatic *APC* mutation in 1 sample. However, Iwama et al. [15] found germline and somatic mutation of APC gene in 2 cases of FAP-associated thyroid carcinomas. These findings suggest that, although somatic mutation of *APC* gene may be seen in some cases of FAP-associated thyroid carcinoma, it is not a required step in the pathogenesis of these neoplasms. However, a number of studies [13, 14, 16] have shown evidence of *ret*/PTC-1 oncogene activation, which is known to be an early molecular event in papillary thyroid carcinoma oncogenesis. This finding would support the concept that FAP-associated thyroid tumors are variants of PTC. 

More recently Xu et al. [17] have demonstrated that aberrant nuclear accumulation of mutant beta-catenin may substitute for *APC* mutations in a subset of CMV of PTC (see Figure 1). It is thought that these sporadic cases are due to a somatic mutation in exon 3 of the beta-catenin gene (*CTNNB1*), further highlighting the analogous role to the APC-beta-catenin pathway. Although only a small number of cases have been studied, the most common carcinogenic genetic abnormality in papillary thyroid carcinoma—BRAF mutation—appears to be absent in FAP-associated thyroid cancer [18].

Thyroid carcinomas occurring in FAP patients have unusual clinical and pathological features, but morphologic and molecular evidence supports the concept that these are variants of PTC. Although the morphologic features are unusual and rarely seen in sporadic PTC, recent reports have emphasized that they are not specific for FAP. Nevertheless, they are sufficiently uncommon that their presence in a thyroid tumor should prompt investigation for FAP. This may be particularly important in the 20% of patients with FAP who have no family history [6].

## Figures and Tables

**Table 1:** Antibody information.

Antibody	Clone	Manufacturer	Retrieval	Dilution
Thyroglobulin	A0251	Dako	Heat	1 : 10.000
EMA	E29	Dako	Heat	1 : 200
Cam 5.2	349205	BD	Protease	1 : 50
Cytokeratin 7	OV-TL 12/30	Dako	Protease	1 : 200
Beta-catenin	Monocl. mouse (IgG)	Cell Marque	Protease	1 : 100

Keratin *Cocktail *				
(i) AE1/AE3	Monocl. mouse	Dako	Protease	1 : 200
(ii) CK wide spectrum screen	Rabbit anticow	Dako	Protease	1 : 1000
(iii) Cam 5.2	349205	BD	Protease	1 : 50

Dako, Mississauga, ON; BD Bioscience, Mississauga, ON; Cell Marque, Rocklin, CA.

## References

[1] Bulow S, Holm NV, Mellemgaard A (1988). Papillary thyroid carcinoma in Danish patients with familial adenomatous polyposis. *International Journal of Colorectal Disease*.

[2] Plail RO, Bussey HJR, Glazer G, Thomson JPS (1987). Adenomatous polyposis: an association with carcinoma of the thyroid. *British Journal of Surgery*.

[3] Harach HR, Williams GT, Williams ED (1994). Familial adenomatous polyposis associated thyroid carcinoma: a distinct type of follicular cell neoplasm. *Histopathology*.

[4] Tomoda C, Miyauchi A, Uruno T (2004). Cribriform-morular variant of papillary thyroid carcinoma: clue to early detection of familial adenomatous polyposis-associated colon cancer. *World Journal of Surgery*.

[5] Ng SB, Sittampalam K, Goh YH, Eu KW (2003). Cribriform-morular variant of papillary carcinoma: the sporadic counterpart of familial adenomatous polyposis-associated thyroid carcinoma. A case report with clinical and molecular genetic correlation. *Pathology*.

[6] Rustgi AK (1994). Hereditary gastrointestinal polyposis and nonpolyposis syndromes. *New England Journal of Medicine*.

[7] Bulow C, Bulow S, Berk T (1997). Is screening for thyroid carcinoma indicated in familial adenomatous polyposis?. *International Journal of Colorectal Disease*.

[8] Giardiello FM, Offerhaus GJA, Lee DH (1993). Increased risk of thyroid and pancreatic carcinoma in familial adenomatous polyposis. *Gut*.

[9] Perrier ND, Van Heerden JA, Goellner JR (1998). Thyroid cancer in patients with familial adenomatous polyposis. *World Journal of Surgery*.

[10] Cetta F, Pelizzo MR, Curia MC, Barbarisi A (1999). Genetics and clinicopathological findings in thyroid carcinomas associated with familial adenomatous polyposis. *American Journal of Pathology*.

[11] Cameselle-Teijeiro J, Chan JKC (1999). Cribriform-morular variant of papillary carcinoma: a distinctive variant representing the sporadic counterpart of familial adenomatous polyposis- associated thyroid carcinoma?. *Modern Pathology*.

[12] Yamashita T, Hosoda Y, Kameyama K, Aiba M, Ito K, Fujimoto Y (1992). Peculiar nuclear clearing composed of microfilaments in papillary carcinoma of the thyroid. *Cancer*.

[13] Cetta F, Olschwang S, Petracci M (1998). Genetic alterations in thyroid carcinoma associated with familial adenomatous polyposis: clinical implications and suggestions for early detection. *World Journal of Surgery*.

[14] Soravia C, Sugg SL, Berk T (1999). Familial adenomatous polyposis-associated thyroid cancer: a clinical, pathological, and molecular genetics study. *American Journal of Pathology*.

[15] Iwama T, Konishi M, Iijima T (1999). Somatic mutation of the APC gene in thyroid carcinoma associated with familial adenomatous polyposis. *Japanese Journal of Cancer Research*.

[16] Cetta F, Curia MC, Montalto G (2001). Thyroid carcinoma usually occurs in patients with familial adenomatous polyposis in the absence of biallelic inactivation of the adenomatous polyposis coli gene. *Journal of Clinical Endocrinology and Metabolism*.

[17] Xu B, Yoshimoto K, Miyauchi A (2003). Cribriform-morular variant of papillary thyroid carcinoma: a pathological and molecular genetic study with evidence of frequent somatic mutations in exon 3 of the *β*-catenin gene. *Journal of Pathology*.

[18] Schuetze D, Hoschar AP, Seethala RR, Assaad A, Zhang X, Hunt JL (2009). The T1799A BRAF mutation is absent in cribriform-morular variant of papillary carcinoma. *Archives of Pathology and Laboratory Medicine*.

